# Complete Genome Sequence of a Strain of *Azospirillum thiophilum* Isolated from a Sulfide Spring

**DOI:** 10.1128/genomeA.01521-15

**Published:** 2016-01-07

**Authors:** Alexey Fomenkov, Tamas Vincze, Margarita Grabovich, Brian P. Anton, Galina Dubinina, Maria Orlova, Elena Belousova, Richard J. Roberts

**Affiliations:** a New England Biolabs, Ipswich, Massachusetts, USA; b Department of Biology, Voronezh State University, Voronezh, Russia; c Winogradsky Institute of Microbiology, Research Center of Biotechnology, Russian Academy of Sciences, Moscow, Russia

## Abstract

We report the complete, closed genome sequence and complete methylome of *Azospirillum thiophilum* strain BV-S^T^.

## GENOME ANNOUNCEMENT

In this work, we report the complete, closed genome sequence of *A. thiophilum* strain BV-S^T^ (DSM 21654^T^, VKM B-2513^T^), which was isolated from a bacterial mat of a sulfide spring. This strain was previously described based on its morphological and biochemical characteristics (1, 2).

The genome was sequenced using the Pacific Biosciences (PacBio RSII) single-molecule real-time (SMRT) sequencing platform. Two SMRTbell libraries were prepared according to modified 10-and 20-kb PacBio sample preparation protocols, including additional separation on a BluePippin DNA size selection system (Sage Science, Beverly, MA) and sequenced using PacBio’s C4 chemistry on 17 SMRT cells using either 180- or 240-min collection times. Sequencing reads were processed, mapped, and assembled by the SMRT analysis pipeline using the HGAP3 protocol and polished using Quiver (3) to give a fully closed genome with 354× coverage. The total genome size was 7,609,460 bp consisting of eight closed chromosomes of 3,037,424, 1,354,909, 878,428, 712,512, 694,460, 518,276, 330,423, and 83,026 bp, respectively, which together encoded a total of 6,620 genes. This multichromosome assembly is consistent with the structure of previously published *Azospirillum* genome sequences (4–6). The assembled sequences were annotated with the NCBI Prokaryotic Genomes Annotation Pipeline (PGAP) and were deposited at DDBJ/EMBL/GenBank.

While this manuscript was in preparation, the same genome was sequenced by another group and the whole-genome shotgun sequence was deposited in GenBank (NZ_LAEL00000000.1) (Y. Kwak and J.-H. Shin, unpublished data). Comparison of the two assemblies using Mauve 2.4.0 indicates the following correspondence between the two sets of contigs: CP012401 and LAEL01000001, with CP012401 2,684 bp larger; LAEL01000011 is completely included within LAEL01000001 and CP012401; CP012402 and LAEL01000002 + LAEL01000008, with CP012402 3,627 bp smaller than those 2 put together; CP012403 and LAEL01000004, with CP012403 446 bp larger; CP012404 and LAEL01000003, with CP012404 4,432 bp larger; CP012405 and LAEL01000007, with CP012405 12,253 bp smaller; CP012406 and LAEL01000005, with CP012406 5,897 bp smaller; CP012407 and LAEL01000006, with CP012407 10,910 bp smaller; CP012408 and LAEL01000009 + LAEL01000010, with CP012408 7,576 bp larger than those 2 put together.

Epigenetic modification at each nucleotide position was determined using kinetic variation (KV) in the nucleotide incorporation rates, and methylated motifs were deduced from the KV data (7–9). Five methylated motifs corresponding to one m4C and four m6A modifications were detected by SMRT sequencing of untreated genomic DNA, and no additional motifs were detected in Tet2 treated DNA, suggesting an absence of m5C modification. The motifs were matched with their corresponding methyltransferase genes, and the results are shown in Table 1. These results have also been deposited in REBASE (10).

**TABLE 1:** Summary of methyltransferases identified in *A. thiophilum* strain BV-S^T^

Motif^*a*^	Assigned or predicted	Methylation type	Restriction modification type
G**A**N**T**C	M.Ath2165I	m6A	II
RG**AT**CY	M.Ath2165II	m6A	II
GGCGC**C**GG		m4C	II
G**A**CCNNNNNNC**T**GG	S.Ath2165ORFDR	m6A	I
GCG**A**TCC		m6A	II

a Modified bases are highlighted in bold.

### Nucleotide sequence accession numbers.

The complete genome sequences of *A. thiophilum* BV-S^T^ (DSM 21654^T^, VKM B-2513^T^) are available in GenBank under the accession numbers CP012401, CP012402, CP012403, CP012404, CP012405, CP012406, CP012407, and CP012408.
